# Expression of RECK and MMP-2 in salivary adenoid cystic carcinoma: Correlation with tumor progression and patient prognosis

**DOI:** 10.3892/ol.2014.1906

**Published:** 2014-02-21

**Authors:** XIAOQING ZHOU, SHENGYUN HUANG, LICHENG JIANG, SHIZHOU ZHANG, WENGANG LI, ZHANWEI CHEN, DONGSHENG ZHANG

**Affiliations:** 1 Department of Oral and Maxillofacial Surgery, First People’s Hospital of Jining, Jining, Shandong 272111, P.R. China; 2 Department of Oral and Maxillofacial Surgery, Shandong Provincial Hospital Affiliated to Shandong University, Jinan, Shandong 250021, P.R. China; 3 Department of Oral and Maxillofacial Surgery, Liaocheng Hospital, Liaocheng, Shandong 252000, P.R. China

**Keywords:** adenoid cystic carcinoma, RECK, MMP-2, prognosis

## Abstract

Reversion-inducing cysteine-rich protein with Kazal motifs (RECK), a glycosylphosphatidylinositol-anchored glycoprotein, inhibits the enzymatic activities of certain matrix metalloproteinases (MMPs). RECK has been studied in numerous human tumors, but the expression of RECK in salivary adenoid cystic carcinoma (SACC), and its correlation with patient prognosis, has never been investigated thus far. In the present study, the expression of RECK and MMP-2 was evaluated in two ACC cell lines and in 83 patients with SACC. The results of quantitative polymerase chain reaction and western blot analysis revealed that the ACC-2 and ACC-M cell lines expressed RECK and MMP-2 mRNA and protein. The immunohistochemical staining in the patients demonstrated that positive expression of RECK and MMP-2 was observed in 21/83 (25.3%) and 69/83 (83.1%) cases, respectively, and that RECK expression was significantly associated with the tumor-node-metastasis stage, histological grade and perineural invasion of patients with SACC (P<0.05). Furthermore, there was a significant association between the positive expression of RECK and that of MMP-2 (P<0.0001). Univariate and multivariate analyses confirmed that a lack of RECK expression was an independent and significant factor for the prediction of a poor prognosis. In conclusion, RECK is a promising prognostic marker and potential therapeutic agent in SACC.

## Introduction

Salivary adenoid cystic carcinoma (SACC) is one of the most common subtypes of malignant tumors occurring in the salivary gland. The characteristics of SACC are the invasion of adjacent tissue and hematogenous spread to the distant organs of the lung, bone and liver (1). Although aggressive surgery has been used, the rate of perineural invasion and distant metastases after 10 years is 50 and 39%, respectively (2). The evolution of SACC is unpredictable in individual patients due to the lack of strong invasion and metastasis indicators, other than advanced stage at presentation. Therefore, the identification of molecular-based specific markers may contribute to prognostic evaluation, by improving our understanding of a single tumor, and more significantly, it may aid biologically-targeted therapy in SACC.

The process of tumor cell invasion into the stromal tissue is closely associated with interactions between the tumor cells and the extracellular matrix (ECM). Altered expression and modification of ECM proteins in tumor cells plays a significant role in their invasion into surrounding normal tissues (3). The matrix metalloproteinase (MMP) family play critical roles in this step by degrading components of the basement membranes and ECM (4). Among the >20 MMPs that have been identified, MMP-2 is a significant proteolytic enzyme (5). MMP-2 can degrade the components of the ECM and basement membrane, destroy the structure of local tissues and increase the ability of vascularization induced by tumors, therefore playing a significant role in all the different stages of the development and invasion of tumors. Thus, the development of novel invasion inhibitors, along with anti-proliferative therapies, may contribute to the control of local tumor growth and tumor spread.

The reversion-inducing cysteine-rich protein with Kazal motifs (RECK) gene was isolated as a transformation suppressor gene; a fibroblast expression library was screened for complementary DNA that was able to induce a non-transformed flat morphology when expressed in v-Ki-ras-transformed NIH3T3 (a mouse embryonic fibroblast cell line) cells (6). RECK is a 110-kDa glycoprotein that is expressed in a number of normal tissues, however it is not expressed in transformed and tumor-derived cells (7). RECK has been demonstrated to inhibit MMP activity through several mechanisms, including the direct inhibition of protease activity, the regulation of cellular release and possible sequestration at the cell surface (8). RECK inhibits the activity of at least three MMP members; MMP-2, MMP-9 and membrane type (MT)1-MMP (9). An increasing amount of evidence has now indicated that the altered expression of RECK is involved in numerous solid tumors, including lung cancer, human breast carcinoma, pancreatic cancer, non-small cell lung cancer and colorectal cancer (10–12). Patients with high RECK protein levels in tumor tissues usually have improved survival outcomes, with tumors being less invasive, as demonstrated in clinical investigations (13). However, our current knowledge of the underlying molecular mechanisms mediated by RECK in SACC is limited. Therefore, we hypothesize that RECK is a novel suppressor gene for invasion and metastasis in SACC, and that it is potentially a good target for SACC therapy.

The purpose of the present study was to examine the expression of RECK and MMP-2 *in vitro* and *in vivo*, and to analyze the correlation between RECK expression and MMP-2 expression in SACC. This study also aimed to clarify the roles of RECK and MMP-2 in the invasion and development of SACC and to explore their role in the prognosis of SACC.

## Materials and methods

### Cell lines

The human SACC cell lines, ACC-2 and ACC-M, were kindly provided by Professor Wantao Chen (Department of Oral and Maxillofacial Surgery, Ninth People’s Hospital, College of stomatology, Shanghai Jiao Tong University, Shanghai, China). The cells were cultured in RPMI 1640 medium supplemented with 10% filtered fetal bovine serum (Hyclone, Logan, UT, USA). The cultures were incubated at 37°C in the humid atmosphere of a 5% CO_2_ incubator.

### RNA isolation and quantitative polymerase chain reaction (qPCR)

Total RNA from the cell lines was obtained using an RNeasy Mini kit (Tiangen Biotech, Beijing, China) according to the manufacturer’s instructions. The quantity and quality of the RNA was assessed by measuring the absorbance at 260 nm and by agarose gel electrophoresis. Total RNA was reverse transcribed with a High Capacity cDNA Archive kit (Takara Bio Inc., Dalian, China), and then an ABI Prism 7000 Sequence Detector (Applied Biosystems, Foster City, CA, USA) was utilized to determine the expression levels of the proteins of interest via qPCR. The thermocycler parameters were 95°C for 15 sec, followed by 45 cycles of 95°C for 5 sec and 60°C for 30 sec. All PCR products were visualized by electrophoresis and ethidium bromide staining in 2% agarose gels. The RECK and MMP-2 primers are shown in Table I.

### Western blot analysis

The cells were harvested, washed twice with cold phosphate-buffered saline (PBS) and lysed in buffer [50 M Tris-HCl (pH 7.4), 150 M NaCl, 2 M EDTA and 1% NP-40], containing protease inhibitors. The protein concentration was quantified using a bicinchoninic acid protein measurement kit (Shenneng Bocai Biology, Co., Ltd., Shanghai, China). A total of 30 μg extract was subjected to SDS-PAGE and then electrophoretically transferred to a nitrocellulose membrane, which was blocked with 5% (w/v) dried skimmed milk-tris-buffered saline and tween 20 [TBST; 10 M Tris-HCl (pH 8.0), 150 M NaCl and 0.05% Tween 20] for 1 h at room temperature. Subsequently, the membrane was incubated for 2 h with antibodies against RECK (Santa Cruz Biotechnologies, Inc., Santa Cruz, CA, USA; at 1:1000 dilution) and MMP-2 (Santa Cruz Biotechnologies, Inc.; at 1:1000 dilution), respectively, in TBST with 5% skimmed milk. Subsequent to being washed in TBST, horseradish peroxidase-conjugated anti-rabbit IgG (Santa Cruz Biotechnology, Inc.) was used as a secondary antibody (1:10,000, in TBST with 2% bovine serum albumin incubated for 1 h). Each sample was also probed with an anti-GAPDH antibody (Santa Cruz Biotechnology, Inc.) as a loading control. Protein bands were visualized by the Alpha Imager 2200 system (Alpha Innotech, San Leandro, CA, USA).

### Patients and specimens

The protocol of the study was approved by the Institutional Ethics Committee of the Provincial Hospital Affiliated to Shandong University (Jinan, China). In total, 83 patients with ACC of the salivary gland were recruited for the study after providing informed consent. The patients underwent resection of their tumors without pre-operative chemotherapy, hormone therapy or radiotherapy at the Department of Oral and Maxillofacial Surgery, Provincial Hospital Affiliated to Shandong University, between May 2001 and March 2010. There were 44 male and 39 female patients aged 19–82 years old (average, 42±12.5 years old) and the tumor sizes ranged between 0.8 and 4.5 cm (median size, 2.1 cm). Of these patients, 36 (43.4%) exhibited stage I and II disease and 47 (56.6%) exhibited stage III and IV disease, according to the American Joint Committee on Cancer staging criteria (14). A total of 27 cases of benign salivary tumors and samples of 16 normal individuals were collected. Pathological slides were reviewed by two pathologists who were not provided with any information on the patients. Each tumor was examined to determine the proportion of tubular, cribiform or solid patterns according to the World Health Organization’s International Histological Classification of Salivary Gland Tumors (15). Three histological grades were determined: Grade I, tumors with tubular and cribriform areas, but without solid components; grade II, cribriform tumors that were either pure or mixed with <30% solid areas; and grade III, tumors with >30% solid patterns.

### Immunohistochemistry

The rabbit polyclonal antibody against RECK (Abcam, 1:100) and the rabbit polyclonal antibody against MMP-2 (Abcam, 1:50) were used for the detection of RECK and MMP-2 in three pathological slides, respectively. Immunohistochemistry was performed on 4-μm thick representative sections of the corresponding formalin-fixed and paraffin-embedded tumor specimens by the streptavidin-peroxidase method following the manufacturer’s instructions (ZSGB-Bio, Beijing, China). Antigen retrieval was accomplished by 0.01 M citrate buffer solution (pH 6.0) for RECK and MMP-2, respectively, in a 700 W microwave oven for 15 min. All stains of RECK and MMP-2 were developed with diaminobenzidine (DAB) for 2 and 3 min, respectively. Negative controls were performed by replacing the primary antibody with PBS.

### Evaluation of immunohistochemical staining

The positive signals of expression of RECK and MMP-2 protein was characterized by the deposition of pale yellow, buff or brown particles; the positive coloration was located in the intracytoplasm. Immunohistochemical staining for RECK and MMP-2 was evaluated in compliance with intensity and proportion. The intensities were scored as follows: 0, no staining; 1, weak staining; 2, moderate staining; and 3, strong staining. The percentage of staining area was classified as: 0, <5% of tumor cells; 1, 6–25% of tumor cells; 2, 26–50% of tumor cells; 3, 51–70% of tumor cells; and 4, >70% of tumor cells. Finally, the two scores were multiplied, providing the final scores: 0–1, negative (−); 2–3, secondary positive (+); and ≥4, positive (++).

### Statistical analysis

All analyses were carried out using the statistical software, SPSS 13.0 (SPSS, Inc., Chicago, IL, USA). The association between RECK and MMP-2 expression and several clinicopathological variables was evaluated according to Pearson’s χ^2^ test or Fisher’s exact test. Survival curves were obtained using the Kaplan-Meier method, and the significance was analyzed by the log-rank test. The effect of variables on survival was assessed using Cox univariate and multivariate regression analyses. The risk ratio and its 95% confidence interval were recorded for each marker. P<0.05 were considered to indicate a statistically significant difference in all of the analyses. Ooverall survival was defined as from the date of surgery to the date a patient succumbed due to SACC.

## Results

### Expression of RECK and MMP-2 mRNA in ACC cell lines

**Figure 1:**
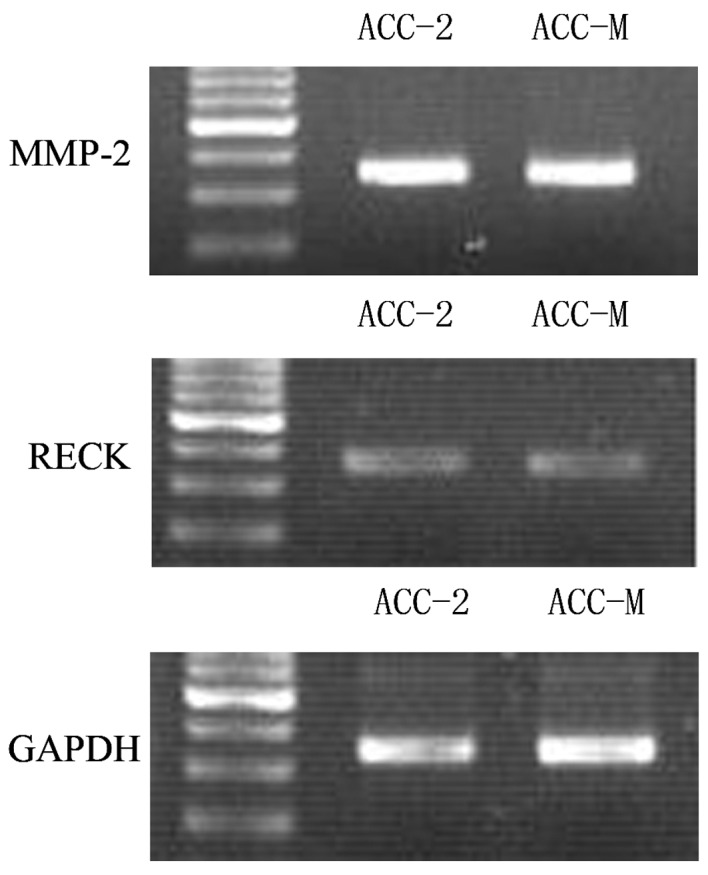
qPCR analysis. Expression of RECK and MMP-2 mRNA in salivary adenoid cystic carcinoma cell lines: ACC-2 and ACC-M. qPCR was performed and PCR product samples were subjected to 2% agarose gel electrophoresis. GAPDH was used as a control. RECK, reversion-inducing cysteine-rich protein with Kazal motifs; MMP-2, matrix metalloproteinase-2; qPCR, qualitative polymerase chain reaction.

To verify whether the mRNA of RECK and MMP-2 was expressed in ACC, the mRNA levels of these two genes were determined in the ACC-2 and ACC-M cell lines by qPCR using specific primers and probes for RECK and MMP-2, with GAPDH as a control. The results revealed RECK and MMP-2 mRNA expression in the two cell lines (Fig. 1).

### Expression of RECK and MMP-2 protein in ACC cell lines

**Figure 2:**
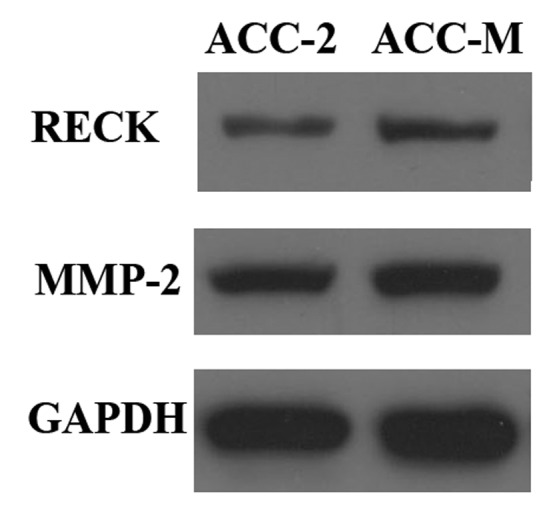
Western blot analysis. Protein expression of RECK and MMP-2 in SACC cell lines, ACC-2 and ACC-M. The total protein of ACC-2 and ACC-M was extracted, and RECK and MMP-2 protein levels were measured by western blotting, respectively. GAPDH was used as a loading control. RECK, reversion-inducing cysteine-rich protein with Kazal motifs; MMP-2, matrix metalloproteinase-2; SACC, salivary adenoid cystic carcinoma.

The protein expression levels of RECK and MMP-2 were determined by western blot analysis in the cell lines, and the GAPDH protein levels were also determined in the same blot to serve as a loading control. These results revealed that RECK and MMP-2 protein was expressed in the ACC-2 and ACC-M cell lines (Fig. 2).

### Expression of RECK and MMP-2 in patients

**Figure 3:**
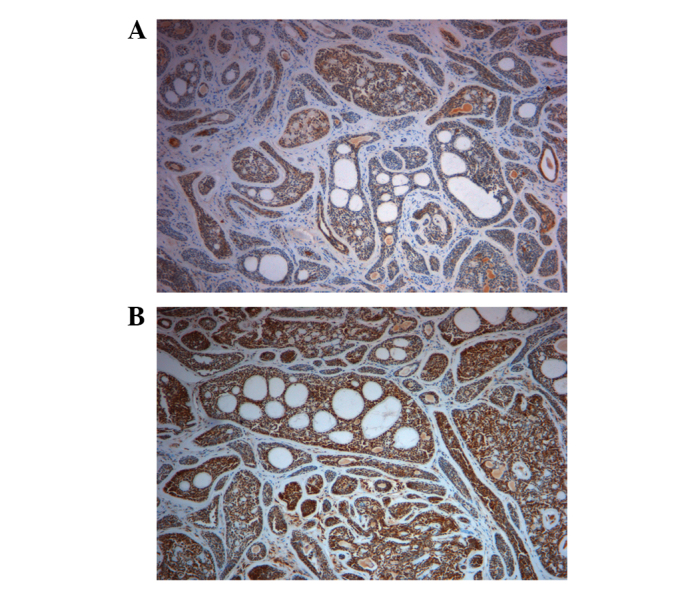
IHC photomicrographs. (A) Positive expression of RECK in salivary adenoid cystic carcinoma (SACC; ×400). (B) Positive expression of MMP-2 in SACC (x400). IHC, immunohistochemistry; RECK, reversion-inducing cysteine-rich protein with Kazal motifs; MMP-2, matrix metalloproteinase-2.

The positive expression of RECK protein was mainly located in the cytoplasm of carcinoma cells, demonstrated by a pale yellow or buff color (Fig. 3A). The percentage of RECK expression increased from the SACC, to the benign salivary tumors, to the normal cases; 25.3% in the SACC, 48.1% in the benign salivary tumors and 87.5% in the normal salivary tissues. The difference in expression rates was considered statistically significant (P<0.05) (Table II). The positive expression of MMP-2 protein was mainly located in the cytoplasm, demonstrated by a buff or brown color (Fig. 3B). The percentage of expression decreased from the SACC, to the benign salivary tumors, to the normal salivary tissues; 83.1% in the ACC, 74.1% in the benign salivary tumors and 25.0% in the normal salivary tissues. The difference of expression rates was considered to be statistically significant (P<0.05) (Table II).

### Correlation between RECK and MMP-2 expression and clinicopathological parameters in SACC patients

Correlations between RECK and MMP-2 expression with various clinicopathological features are summarized in Table III. No significant correlations were observed between RECK and MMP-2 expression, and age, tumor size and gender. RECK expression was significantly associated with tumor-node-metastasis (TNM) stage (P=0.047), perineural invasion (P=0.019) and histological grade (P=0.006). However, no significant correlations were revealed between MMP-2 expression and histological grade (P=0.064).

### Correlation of RECK and MMP-2 in SACC

As revealed in Table IV, the MMP-2 expression rate was 45.5% (10/22) in tissues with positive (+ and ++) RECK expression. In tissues negative (−) for RECK expression, the MMP-2 expression rate was 95.2% (59/62). Pearson’s χ^2^ test revealed that there was a significant negative correlation between the expression of RECK and MMP-2 (χ^2^, 38.202; P<0.0001).

### Overall survival analysis

**Figure 4:**
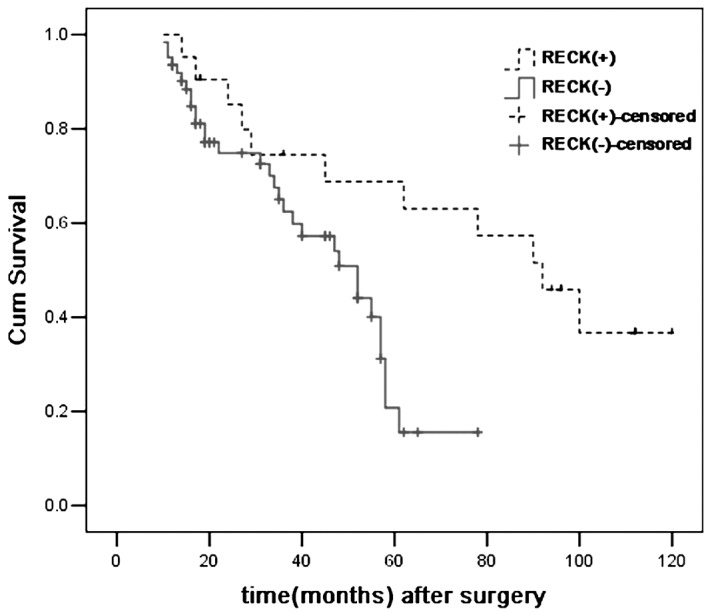
Kaplan-Meier survival curves for cumulative survival rate of SACC patients according to RECK expression (P=0.004). The solid line represents a negative staining result, and the dotted line represents a positive staining result. RECK, reversion-inducing cysteine-rich protein with Kazal motifs; MMP-2, matrix metalloproteinase-2.

The average follow-up period was 54 months (range, 10–120 months). In total, 42 patients (50.6%)succumbed to SACC, 5 patients (6.0%) succumbed during the follow-up period, 4 patients (4.8%) succumbed to an unrelated cause and 32 patients (38.6%) remained alive on the day of the study. The survival curves demonstrated that the overall survival in the RECK-positive expression group was improved in comparison to the RECK-negative expression group (P=0.009), based on the use of the Kaplan-Meier method and log-rank test (Fig. 4). As summarized in Table V, the univariate analysis revealed that the following variables were significantly associated with the prognosis, including RECK and MMP-2 expression, histological grade, TNM stage and perineural invasion, while the multivariate analysis revealed that RECK expression and histological grade also had an independent prognostic effect on the overall survival of the SACC patients.

## Discussion

In the present study, the expression of RECK and MMP-2 mRNA and protein was examined first *in vitro* using two human ACC cell lines. The results revealed the positive expression of RECK in the ACC-2 and ACC-M cell lines. Therefore, the *in vivo* expression of RECK and MMP-2 in SACC was investigated using an immunohistochemistry assay. First, the positive expression of RECK was observed in 21/83 (25.3%) of SACC cases, and RECK expression was significantly associated with the TNM stage, histological pattern and perineural invasion of patients with SACC (P<0.05). Furthermore, there was a significant inverse correlation between RECK-positive expression and MMP-2-positive expression (P=0.046), and the RECK expression was significantly associated with overall survival.

Recurrence and metastasis is responsible for patient mortality in most solid tumors (16–18). Degradation and remodeling of the ECM are necessary steps in tumor development. All ECM components can be degraded by MMPs, and each ECM element is cleaved by a specific MMP or MMP group. In particular, MMP-2 is upregulated in SACC and a number of other malignant tumors, and contributes to the invasion of tumor cells by degrading the ECM (19–21). RECK, initially identified as a transformation suppressor gene, is normally expressed in adult human tissues and is downregulated in numerous solid tumors, including pancreatic cancer, non-small cell lung cancer and colorectal cancer (11,12,22). Similarly, the present results revealed the mRNA and protein expression of RECK and MMP-2 in human ACC cell lines, and identified that RECK expression was significantly lower in SACC than in normal tissues.

Previous studies (23–25) have reported that RECK function is correlated with the inhibition of MMP-2, MMP-9 and MT1-MMP. Masui *et al* (26) reported a significant inverse correlation between RECK and MMP-2 expression in pancreatic cancer. To explore the correlation between MMP-2 and RECK in SACC in the present study, 83 specimens of SACC were collected to examine the expression of MMP-2 and RECK by immunohistochemistry assay. There was a significant negative correlation between the expression of RECK and MMP-2 (P<0.0001), and the result was consistent with the aforementioned study. Furthermore, the correlation between RECK expression and various clinicopathological features was evaluated, revealing that RECK expression was significantly associated with histological grade, TNM stage and the perineural invasion of patients with SACC (P<0.05), but that MMP-2 expression had no significant association with histological grade (P=0.064); RECK was possibly functioning as more than an MMP inhibitor, and further studies are therefore required in this area.

Furumoto *et al* (27) reported that high RECK expression correlates with less invasive tumors and an improved prognosis in patients with hepatocellular carcinoma, and that downregulated RECK expression could induce tumor angiogenesis and promote tumor regression. Masui *et al* (26) examined RECK expression in pancreatic cancer and reported that tumors with positive RECK staining were significantly less invasive in comparison to RECK-negative tumors, indicating the potential value of RECK as a prognostic molecular marker for pancreatic cancer. In the present study, the multivariate survival analysis revealed that RECK expression (P=0.014) was significantly associated with an improved survival rate and could be an independent prognostic indicator in SACC. In addition, histological grade (I, II/III; P=0.038) appeared to be the significantly independent clinicopathological factor involved with overall survival, which has been revealed to indicate a poor prognosis for SACC in a previous study (28). Taken together, these findings indicate that RECK may serve as a favorable prognostic predictor in SACC, and increase the ability to predict patient prognosis when used in combination with histological grade (I, II/III).

An increase in RECK expression appears to be a reasonable treatment strategy to enhance the overall survival rate of SACC patients, and several ways to upregulate RECK expression have been suggested. Yan *et al* (29) reported that tomatidine inhibits the invasion of human lung adenocarcinoma A549 cells by reducing MMP-2 expression and enhancing RECK expression. Hsu *et al* (7) reported that erbB2 transcriptionally represses RECK expression through HDAC1 and Sp1, and demonstrated that the histone deacetylase inhibitor, trichostatin A, upregulates RECK expression *in vitro*. Studies enhancing RECK expression by drug or gene knock out *in vitro* and *in vivo*, in order to examine the effect on the migration and invasion of SACC, are required.

In conclusion, the present data indicates that RECK was discovered as a novel molecular biomarker and may actively be involved in the progression, invasion and metastasis of SACC. RECK may reduce tumor progression through functionally downregulating MMP-2 expression. Measurements of the expression of RECK and MMP-2 are valuable, not only to assess patient prognosis, but to also develop new strategies for cancer prevention and therapeutic intervention.

## Figures and Tables

**Table I:** Bases of primers for qPCR.

Gene	Primer
RECK	F: 5′-TGCAAGCAGGCATCTTCAAA-3′
	R: 5′-ACCGAGCCCATTTCATTTCTG-3′
MMP-2	F: 5′-AGCTCCCGGAAAAGATTGATG-3′
	R: 5′-CAGGGTGCTGGCTGAGTAGAT-3′
GAPDH	F: 5′-GCAGGGGGGAGCCAAAAGGG-3′
	R: 5′-TGCCAGCCCCAGCGTCAAAG-3′

qPCR, quantitative polymerase chain reaction; RECK, reversion-inducing cysteine-rich protein with Kazal motifs; MMP-2, matrix metalloproteinase-2.

**Table II:** Expression of RECK and MMP-2 in SACC, benign salivary tumors and normal cases.

Group	Total (n)	RECK				χ^2^	P-value	MMP-2				χ^2^	P-value
	
		− (n)	+ (n)	++ (n)	PP (%)			− (n)	+ (n)	++ (n)	PP (%)		
SACC	83	62	9	12	25.3	25.007	0.000	14	29	40	83.1	25.405	<0.0001
BST	27	14	7	6	48.1			7	9	11	74.1		
NC	16	2	5	9	87.5			12	4	0	25.0		

ACC, adenoid cystic carcinoma; BST, benign salivary tumors; NC, normal cases; PP, percentage of positive; RECK, reversion-inducing cysteine-rich protein with Kazal motifs; MMP-2, matrix metalloproteinase-2.

**Table III:** Clinicopathological features of the SACC patients and their primary tumors and their association with RECK and MMP-2 expression.

		RECK					MMP-2				
							
Factor	No. of cases	− (n)	+ (n)	PP (%)	χ^2^	P-value	− (n)	+ (n)	PP (%)	χ^2^	P-value
Gender											
Female	34	25	9	26.5	0.042	0.838	5	29	85.3	0.192	0.661
Male	49	37	12	24.5			9	40	81.6		
Age, years											
≥52	43	32	11	25.6	0.004	0.951	8	35	81.4	0.192	0.661
<52	40	30	10	25.0			6	34	85.0		
Tumor size, cm											
3	51	39	12	23.5	0.220	0.639	10	41	80.4	0.708	0.400
≥3	32	23	9	28.1			4	28	87.5		
TNM stage											
I, II	36	23	13	36.1	3.931	0.047^a^	10	26	72.2	5.397	0.020^a^
III, IV	47	39	8	17.0			4	43	91.5		
Histological grade											
I	36	21	15	41.7	10.17	0.006^a^	10	26	72.2	5.489	0.064
II	19	15	4	21.1			2	17	89.5		
III	28	26	2	7.1			2	26	92.9		
Perineural invasion											
No	45	29	16	35.6	5.469	0.019^a^	11	34	75.6	4.024	0.045^a^
Yes	38	33	5	13.2			3	35	92.1		

a Significantly different by χ^2^ test or Fisher’s exact test.

PP, percentage of positive; RECK, reversion-inducing cysteine-rich protein with Kazal motifs; MMP-2, matrix metalloproteinase-2; SACC, salivary adenoid cystic carcinoma; TNM, tumor-node-metastasis.

**Table IV:** Analysis of the correlation between the expression of RECK and MMP-2 in SACC.

RECK	Total, n	MMP-2 (n)			χ^2^	P-value

		−	+	++		
−	62	3	22	37	38.202	<0.0001^a^
+	9	3	4	2		
++	12	9	3	0		

a Significantly different by χ^2^ test.

RECK, reversion-inducing cysteine-rich protein with Kazal motifs; MMP-2, matrix metalloproteinase-2; SACC, salivary adenoid cystic carcinoma.

**Table V:** Prognostic factors in the Cox proportional hazards model.

	Univariate analysis		Multivariate analysis	
		
Variables	HR (95% CI)	P-value	HR (95% CI)	P-value
Gender (female/male)	0.85 (0.28–1.47)	0.561	nd	nd
Age (<52 years/≥52 years)	1.69 (0.38–2.79)	0.476	nd	nd
TNM stage (I, II/III, IV)	3.26 (1.96–6.12)	0.038^a^	2.01 (1.26–3.58)	0.296
Tumor size (≤3 cm/>3 cm)	2.98 (1.26–5.13)	0.233	nd	nd
Histological grade (I, II/III)	3.65 (1.49–5.23)	0.007^a^	3.16 (1.27–5.79)	0.038^b^
RECK expression (positive/negative)	2.45 (0.69–4.56)	0.009^a^	4.57 (0.85–24.55)	0.014^b^
MMP-2 expression (positive/negative)	4.26 (2.13–6.39)	0.021^a^	1.69 (0.48–4.57)	0.079
Perineural invasion (positive/negative)	3.19 (1.26–4.81)	0.067	nd	nd

HR, hazard ratio; CI, confidence interval; nd, not done; TNM, tumor-node-metastasis; RECK, reversion-inducing cysteine-rich protein with Kazal motifs; MMP-2, matrix metalloproteinase-2.

a P<0.05 by univariate analysis.

b P<0.05 by multivariate analysis.

## References

[1] van der Wal JE, Becking AG, Snow GB, van der Waal I (2002). Distant metastases of adenoid cystic carcinoma of the salivary glands and the value of diagnostic examinations during follow-up. Head Neck.

[2] Terhaard CH, Lubsen H, Van der Tweel I (2004). Salivary gland carcinoma: independent prognostic factors for locoregional control, distant metastases and overall survival: results of the Dutch head and neck oncology cooperative group. Head Neck.

[3] Honma K, Miyata T, Ochiya T (2007). Type I collagen gene suppresses tumor growth and invasion of malignant human glioma cells. Cancer Cell Int.

[4] López-Otín C, Matrisian LM (2007). Emerging roles of proteases in tumour suppression. Nat Rev Cancer.

[5] Visse R, Nagase H (2003). Matrix metalloproteinases and tissue inhibitors of metalloproteinases: structure, function, and biochemistry. Circ Res.

[6] Takahashi C, Sheng Z, Horan TP (1998). Regulation of matrix metalloproteinase-9 and inhibition of tumor invasion by the membrane-anchored glycoprotein RECK. Proc Natl Acad Sci USA.

[7] Hsu MC, Chang HC, Hung WC (2006). HER-2/neu represses the metastasis suppressor RECK via ERK and Sp transcription factors to promote cell invasion. J Biol Chem.

[8] Welm B, Mott J, Werb Z (2002). Developmental biology: vasculogenesis is a wreck without RECK. Curr Biol.

[9] Sasahara RM, Brochado SM, Takahashi C (2002). Transcriptional control of the RECK metastasis/angiogenesis suppressor gene. Cancer Detect Prev.

[10] Takagi S, Simizu S, Osada H (2009). RECK negatively regulates matrix metalloproteinase-9 transcription. Cancer Res.

[11] Takenaka K, Ishikawa S, Yanagihara K (2005). Prognostic significance of reversion-inducing cysteine-rich protein with Kazal motifs expression in resected pathologic stage IIIA N2 non-small-cell lung cancer. Ann Surg Oncol.

[12] Span PN, Sweep CG, Manders P, Beex LV, Leppert D, Lindberg RL (2003). Matrix metalloproteinase inhibitor reversion-inducing cysteine-rich protein with Kazal motifs: a prognostic marker for good clinical outcome in human breast carcinoma. Cancer.

[13] Kang HG, Kim HS, Kim KJ, Oh JH, Lee MR, Seol SM, Han I (2007). RECK expression in osteosarcoma: correlation with matrix metalloproteinases activation and tumor invasiveness. J Orthop Res.

[14] Ko YH, Roh SY, Won HS (2010). Prognostic significance of nuclear survivin expression in resected adenoid cystic carcinoma of the head and neck. Head Neck Oncol.

[15] Seifert G, Sobin LH (1992). The World Health Organization’s Histological Classification of Salivary Gland Tumors. A commentary on the second edition. Cancer.

[16] Lee JH, Welch DR (1997). Suppression of metastasis in human breast carcinoma MDA-MB-435 cells after transfection with the metastasis suppressor gene, KiSS-1. Cancer Res.

[17] Welch DR, Steeg PS, Rinker-Schaeffer CW (2000). Molecular biology of breast cancer metastasis. Genetic regulation of human breast carcinoma metastasis. Breast Cancer Res.

[18] Yang J, Mani SA, Donaher JL (2004). Twist, a master regulator of morphogenesis, plays an essential role in tumor metastasis. Cell.

[19] Shirasuna K, Saka M, Hayashido Y, Yoshioka H, Sugiura T, Matsuya T (1993). Extracellular matrix production and degradation by adenoid cystic carcinoma cells: participation of plasminogen activator and its inhibitor in matrix degradation. Cancer Res.

[20] Vihinen P, Kähäri VM (2002). Matrix metalloproteinases in cancer: prognostic markers and therapeutic targets. Int J Cancer.

[21] Freitas VM, Vilas-Boas VF, Pimenta DC (2007). SIKVAV, a laminin alpha1-derived peptide, interacts with integrins and increases protease activity of a human salivary gland adenoid cystic carcinoma cell line through the ERK 1/2 signaling pathway. Am J Pathol.

[22] Takeuchi T, Hisanaga M, Nagao M (2004). The membrane-anchored matrix metalloproteinase (MMP) regulator RECK in combination with MMP-9 serves as an informative prognostic indicator for colorectal cancer. Clin Cancer Res.

[23] Oh J, Takahashi R, Kondo S (2001). The membrane-anchored MMP inhibitor RECK is a key regulator of extracellular matrix integrity and angiogenesis. Cell.

[24] Sternlicht MD, Werb Z (2001). How matrix metalloproteinases regulate cell behavior. Annu Rev Cell Dev Biol.

[25] Sasahara RM, Takahashi C, Noda M (1999). Involvement of the Sp1 site in ras-mediated downregulation of the RECK metastasis suppressor gene. Biochem Biophys Res Commun.

[26] Masui T, Doi R, Koshiba T (2003). RECK expression in pancreatic cancer: its correlation with lower invasiveness and better prognosis. Clin Cancer Res.

[27] Furumoto K, Arii S, Mori A (2001). RECK gene expression in hepatocellular carcinoma: correlation with invasion-related clinicopathological factors and its clinical significance. Reverse-inducing-cysteine-rich protein with Kazal motifs. Hepatology.

[28] Yang X, Dai J, Li T (2010). Expression of EMMPRIN in adenoid cystic carcinoma of salivary glands: correlation with tumor progression and patients’ prognosis. Oral Oncol.

[29] Yan KH, Lee LM, Yan SH, Huang HC, Li CC, Lin HT, Chen PS (2013). Tomatidine inhibits invasion of human lung adenocarcinoma cell A549 by reducing matrix metalloproteinases expression. Chem Biol Interact.

